# Supplementary material for: Segregated oceanic crust trapped at the bottom mantle transition zone revealed from ambient noise interferometry
Source: Nat Commun. 2021 May 5;12:2531. doi: 10.1038/s41467-021-22853-2 (PMC8099894; doi:10.1038/s41467-021-22853-2)
Supplement: Supplementary file 1 — Supplementary Information [file 41467_2021_22853_MOESM1_ESM.pdf]

Supplementary Information for

Segregated oceanic crust trapped at the bottom mantle transition zone revealed from ambient noise interferometry

Jikun Feng[1,2*], Huajian Yao[1,2,3*], Yi Wang[1,2], Piero Poli[4], Zhu Mao[1,2].

Affiliations:

[1] Laboratory of Seismology and Physics of Earth's Interior, School of Earth and Space Sciences, University of Science and Technology of China, Hefei, China.

[2] CAS Center for Excellence in Comparative Planetology, University of Science and Technology of China, Hefei, China.

[3] Mengcheng National Geophysical Observatory, University of Science and Technology of China, Mengcheng, China.

[4] University Grenoble Alpes, CNRS, ISTerre, Grenoble, France.

Correspondence to: jkfeng@mail.ustc.edu.cn (J. Feng) or hjyao@ustc.edu.cn (H.Yao)

This PDF file includes:

Supplementary Figs. 1 to 13

Supplementary Notes 1 to 4

Supplementary References

Supplementary Figures

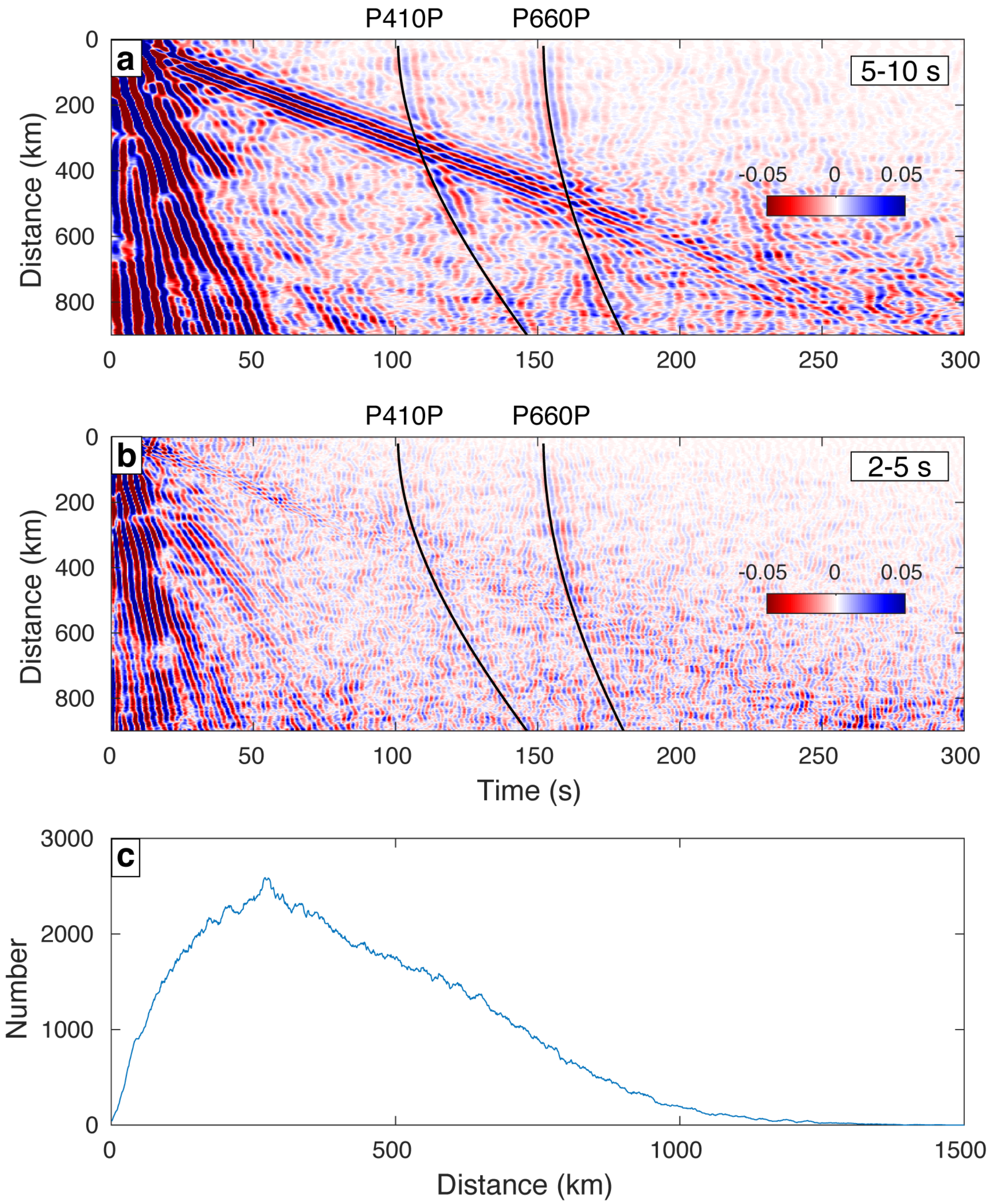

Supplementary Fig. 1. Filtered ambient noise cross-correlations and their distribution. All the ambient noise cross-correlation functions (NCFs) were filtered into 5-10 s (**a**) and 2-5 s (**b**) period bands and stacked within a series of overlapped distance bins, where the width of bins is

set to 19 km. The stacked waveforms were normalized by the number of stacked NCFs within each bin. Black lines denote the theoretical traveltime curves of P410P and P660P. **c** The number of stacked NCFs within each distance bin.

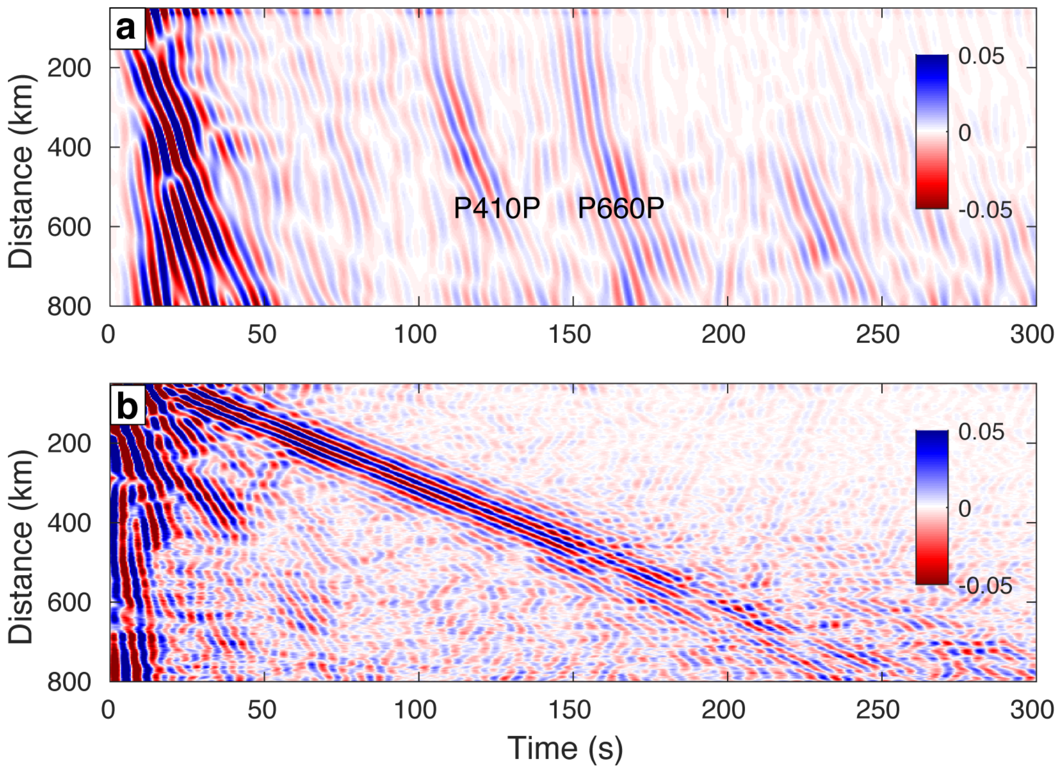

Supplementary Fig. 2. Separation of Rayleigh waves and reflected body waves. **a** Separated reflected body waves within the 5-10 s period band. **b** Separated Rayleigh waves within the 5-10 s period band . All the ambient noise cross-correlations functions (NCFs) were first regularized by stacking within a series of overlapped 19-km wide distance bins and normalized by the number of stacked NCFs within each bin (Supplementary Fig. 1c) before separation.

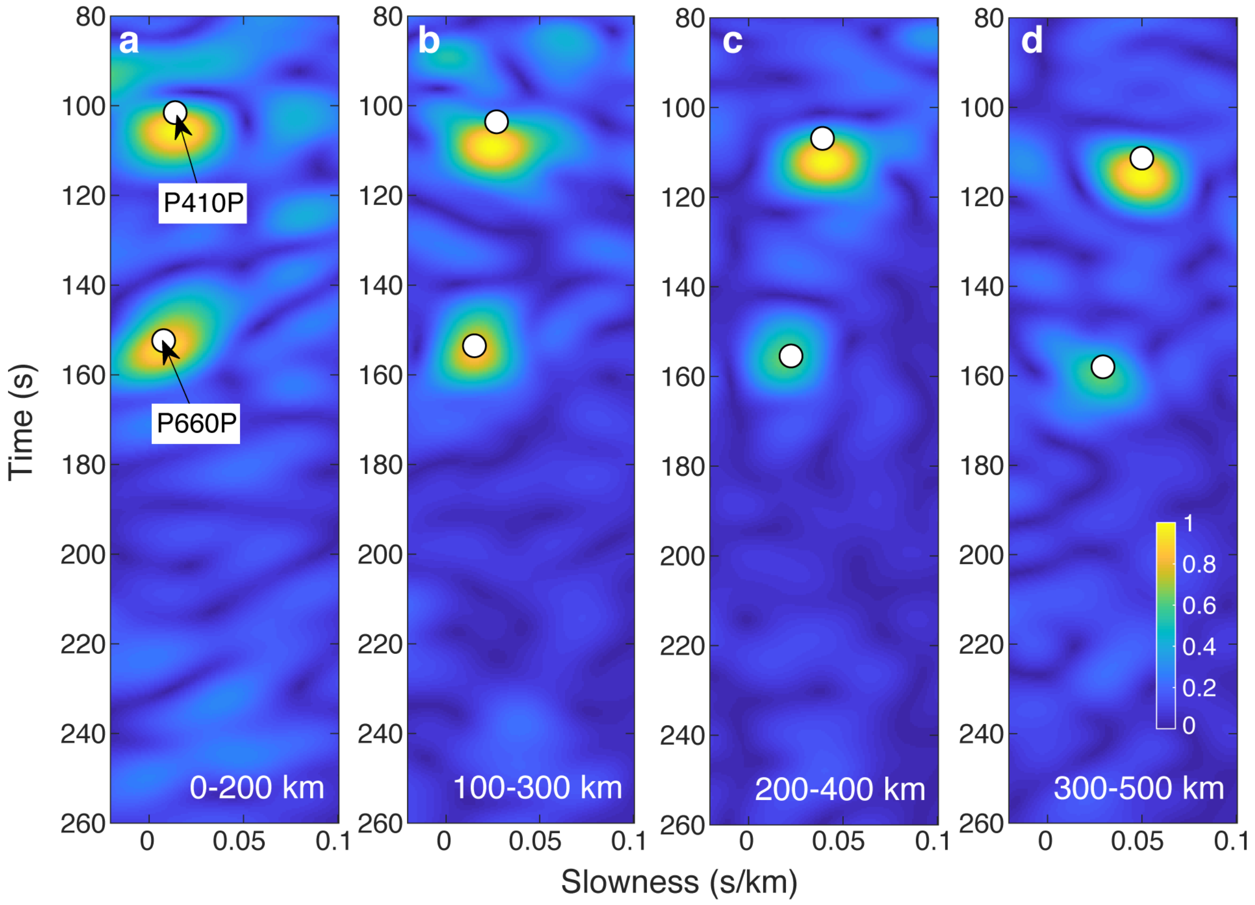

Supplementary Fig. 3. Slant stacking of all the ambient noise cross-correlations. Four panels exhibit the normalized envelope of the time-slowness slant stacking of the ambient noise cross-correlations performed within four distance bins: 0-200 km, 100-300 km, 200-400 km, and 300-500 km. The ambient noise cross-correlations were filtered to 5-10 s before slant stacking. Red background color indicates strong stacking power. The white circles indicate the theoretical arrival times and ray parameters of P410P and P660P phases at 100 km, 200 km, 300 km, and 400 km epicenter distances calculated with the Taup toolkit[1] from the ak135-f model[2].

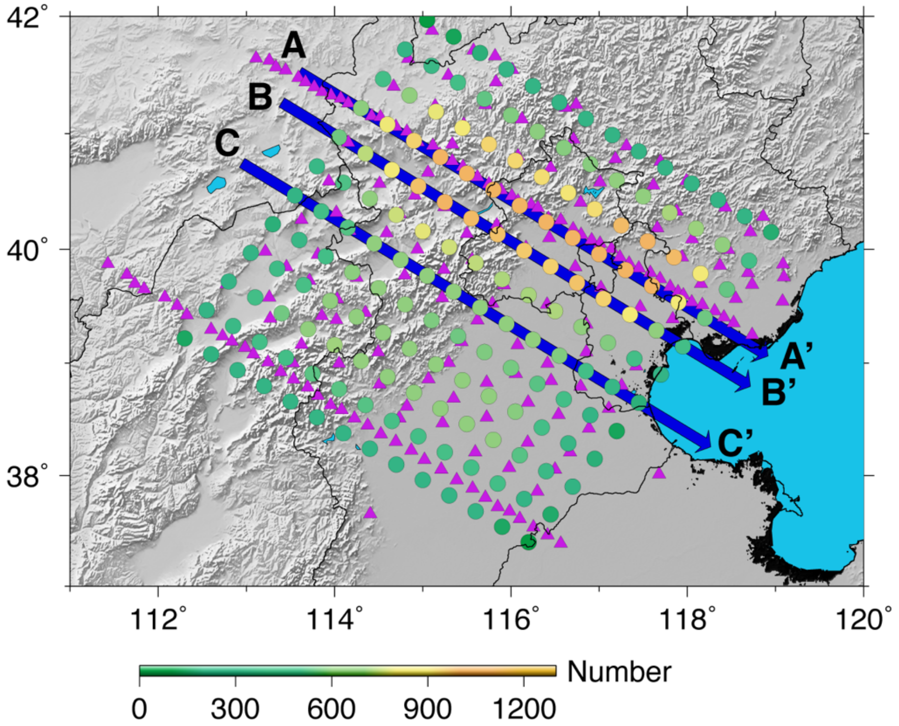

Supplementary Fig. 4. Distribution of stations, stacking bins and profiles. Purple triangles represent the dense seismic array stations. Colored circles denote stacking bin centers. Selected three profiles are indicated by blue arrowed lines labeled as AA', BB', and CC'. The color of each circle depicts the number of stacked ambient noise cross-correlation functions within the corresponding bin.

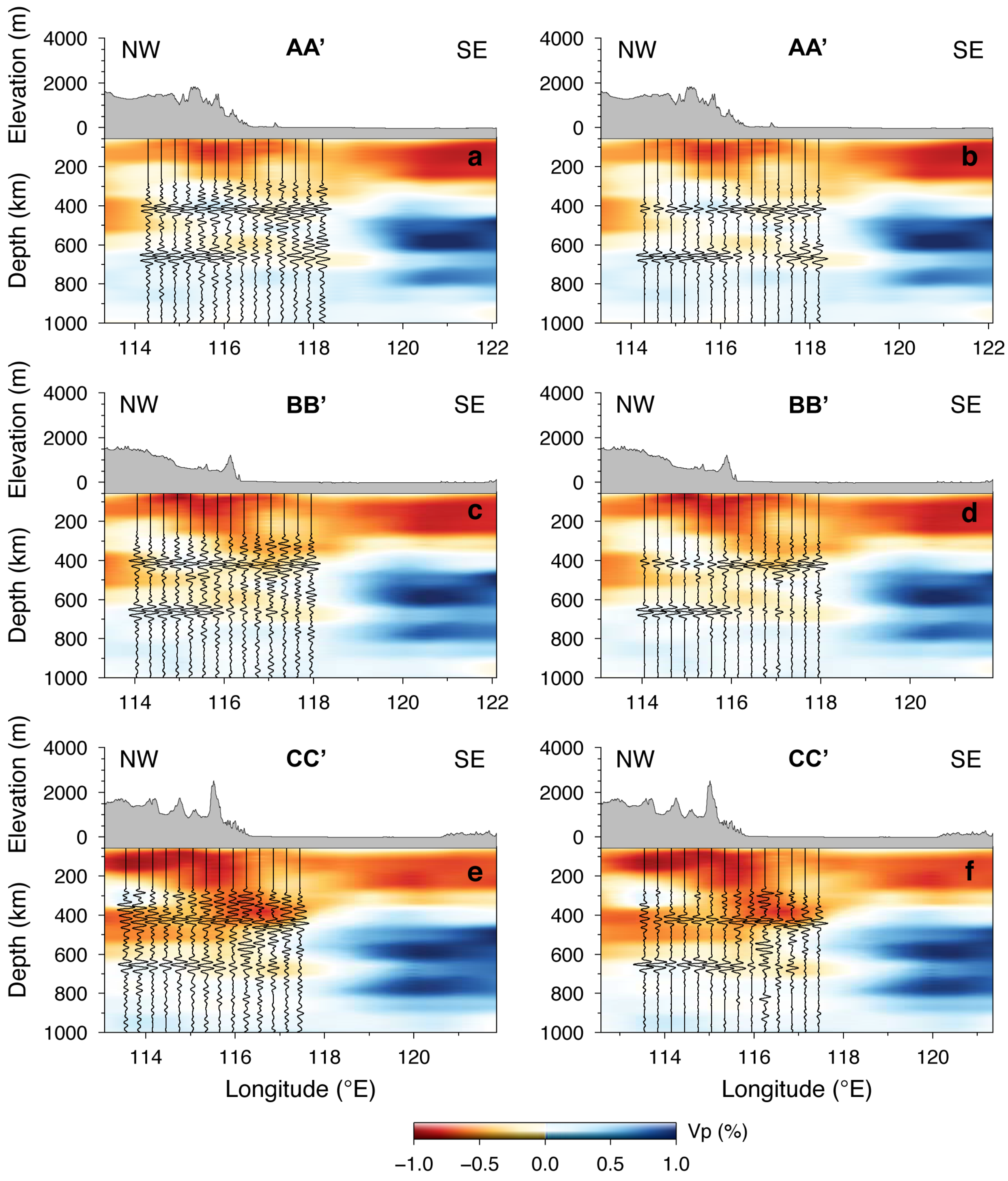

Supplementary Fig. 5. Common reflection point stacking results. The reflected body waveforms (black traces), filtered to 5-10 s, along three selected profiles shown in Supplementary Fig. 4. The images on the left column (**a**, **c**, and **e**) show the linear stacking results. The images on

the right column (**b**, **d**, and **f**) show the phase-weighted stacking results. The background color of each image shows P-wave speed perturbation from ref[3]. The topography along each profile is also plotted above each image.

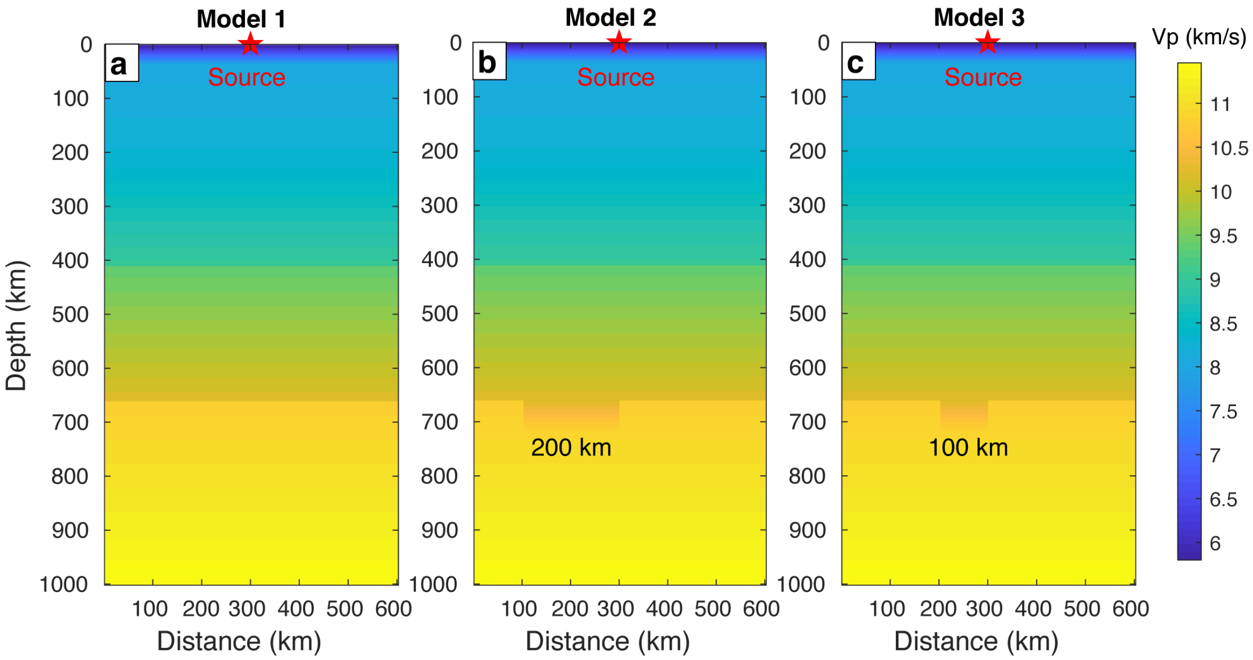

Supplementary Fig. 6 Designed models to test the resolution of the P660P phase. **a** A simple model derived from iasp91 model[4] with sharp 410- and 660-km discontinuities. **b** A model similar to **a** but with a 200-km wide gradual 660-km discontinuity (a linear increase from 660 km to 720 km). **c** A model similar to **b** but with a 100-km wide gradual 660-km discontinuity. Vertical force sources are set on the free surface at 300 km to excite seismic wavefield.

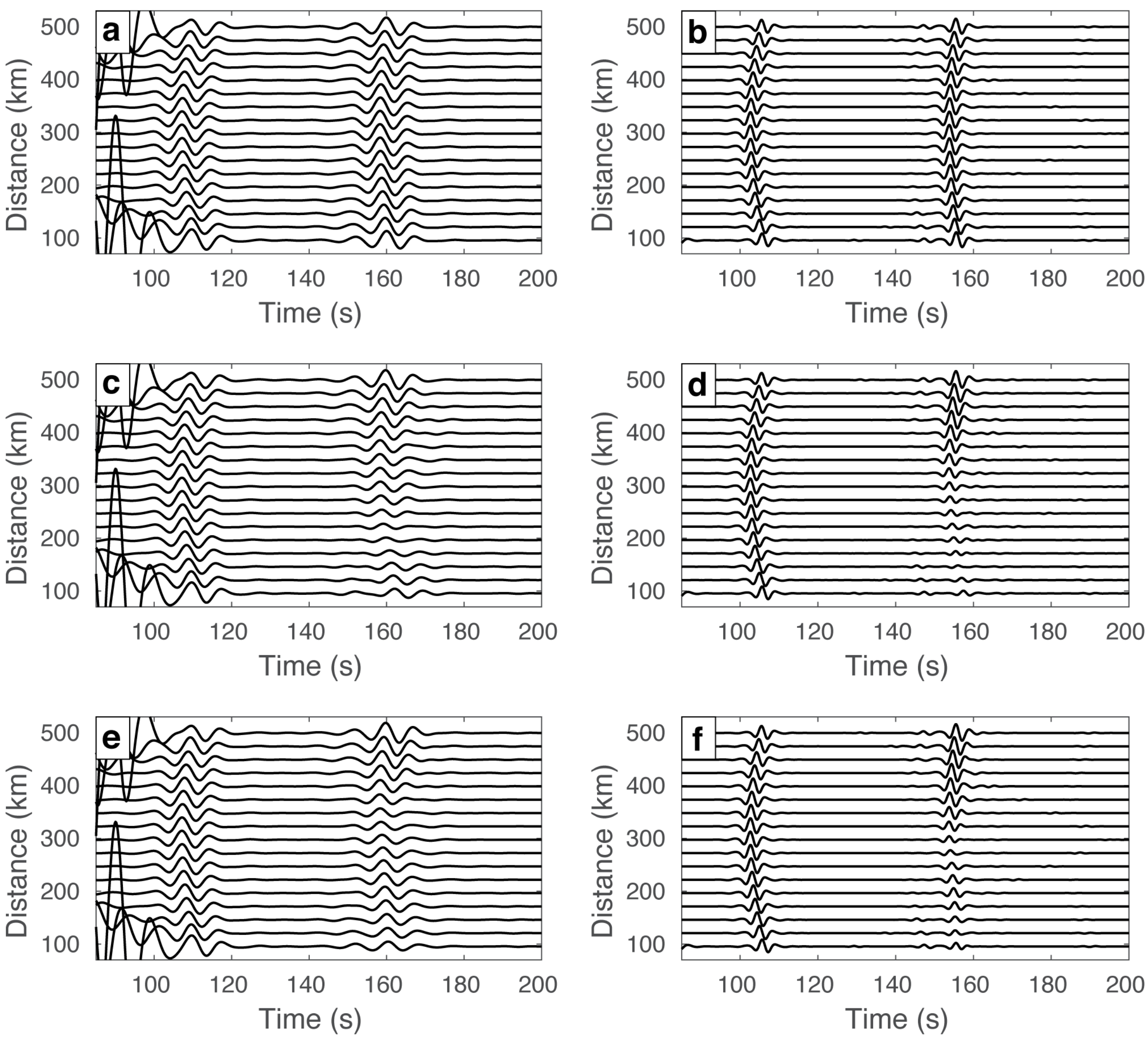

Supplementary Fig. 7 Synthetic waveforms calculated from three designed models. Seismic waveforms recorded on the free surface were calculated by the SPECFEM2D package[5]. **a** and **b** show the synthetic waveforms from Model 1 filtered to 2-5 s and 5-10 s, respectively. **c** and **d** show the synthetic waveforms from Model 2 filtered to two target period bands. **e** and **f** show the synthetic waveforms from Model 3 filtered to two target period bands.

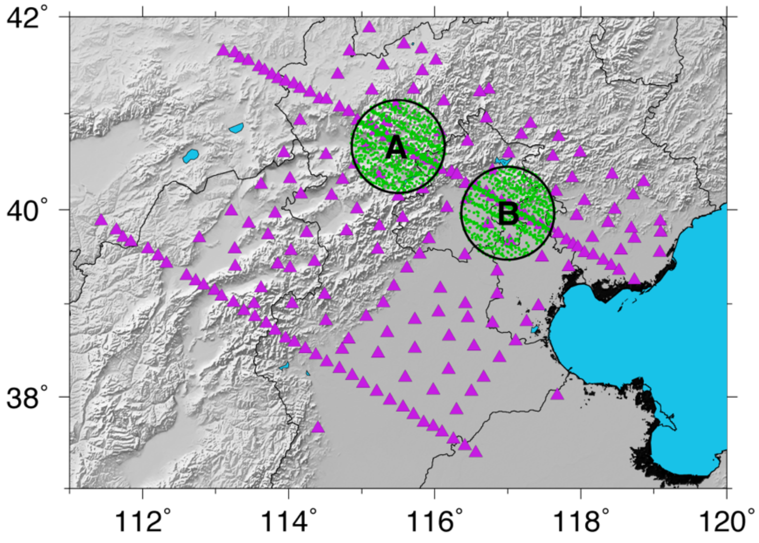

Supplementary Fig. 8. The dense portable seismic array and two example bins. Purple triangles represent the portable array stations. Two example bins are labeled as A and B, respectively. The green dots represent reflection points at the mantle transition zone interfaces of selected station pairs.

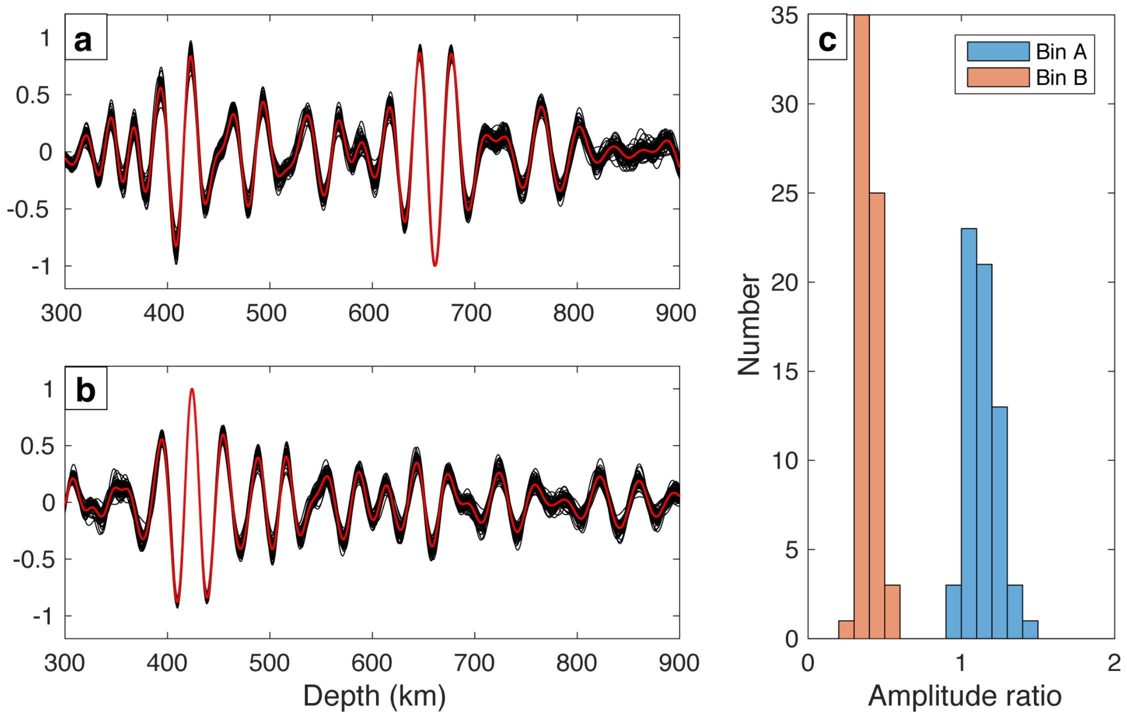

Supplementary Fig. 9. Reliability test of the amplitude ratio of reflected phases. **a** Bootstrap test within bin A. Black traces represent the randomly stacked results within the 5-10 s period band and the red trace is the average of all available daily ambient noise cross-correlations. **b** Bootstrap test within bin B. **c** The amplitude ratio (Amp_{P660P}/Amp_{P410P}) distribution of the randomly stacked traces from bin A and bin B (blue and orange bars, respectively).

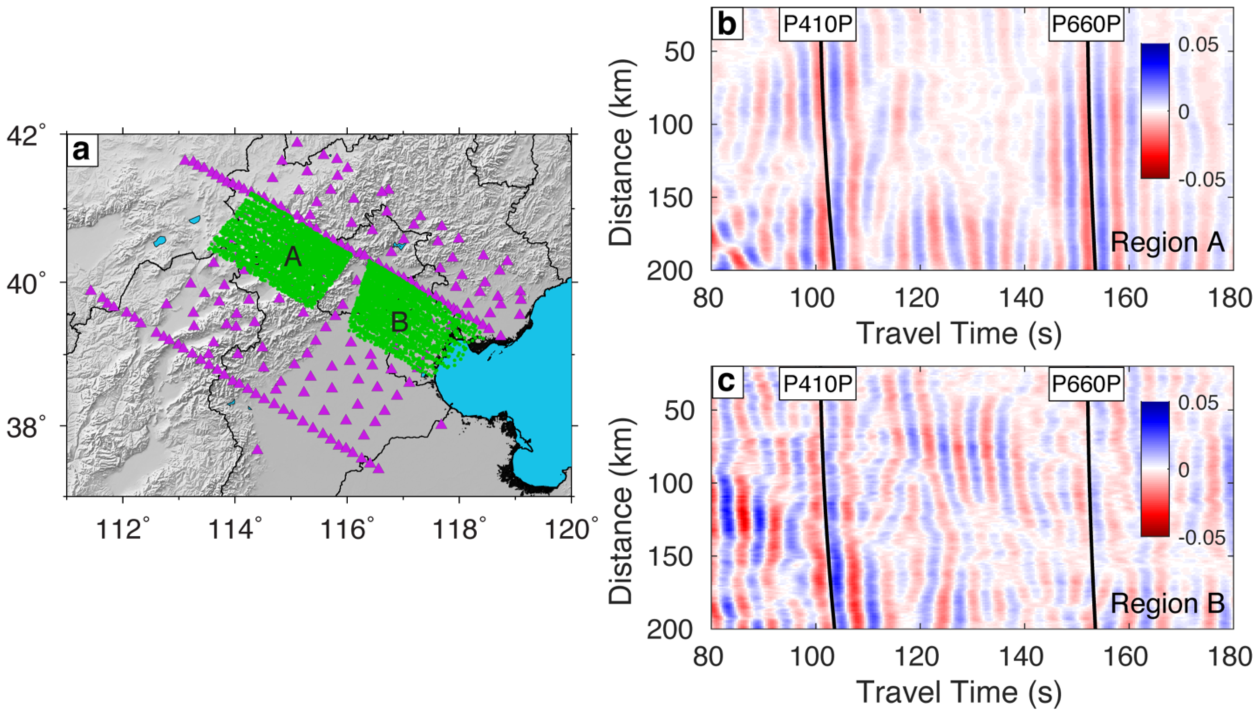

Supplementary Fig. 10 Comparison of reflected waveforms from two independent regions. **a** Station map and the definition of two independent regions (A and B). Green dots denote the reflection points of ambient noise cross-correlation functions (NCFs). **b** and **c** display all the NCFs reflected within regions A and B, respectively. The NCFs were filtered to 5-10 s and stacked within a series of overlapped distance bins, and the width of bins is set to 37 km. The stacked waveforms were normalized by the number of stacked NCFs within each distance bin. Black lines denote the theoretical traveltime curves of P410P and P660P.

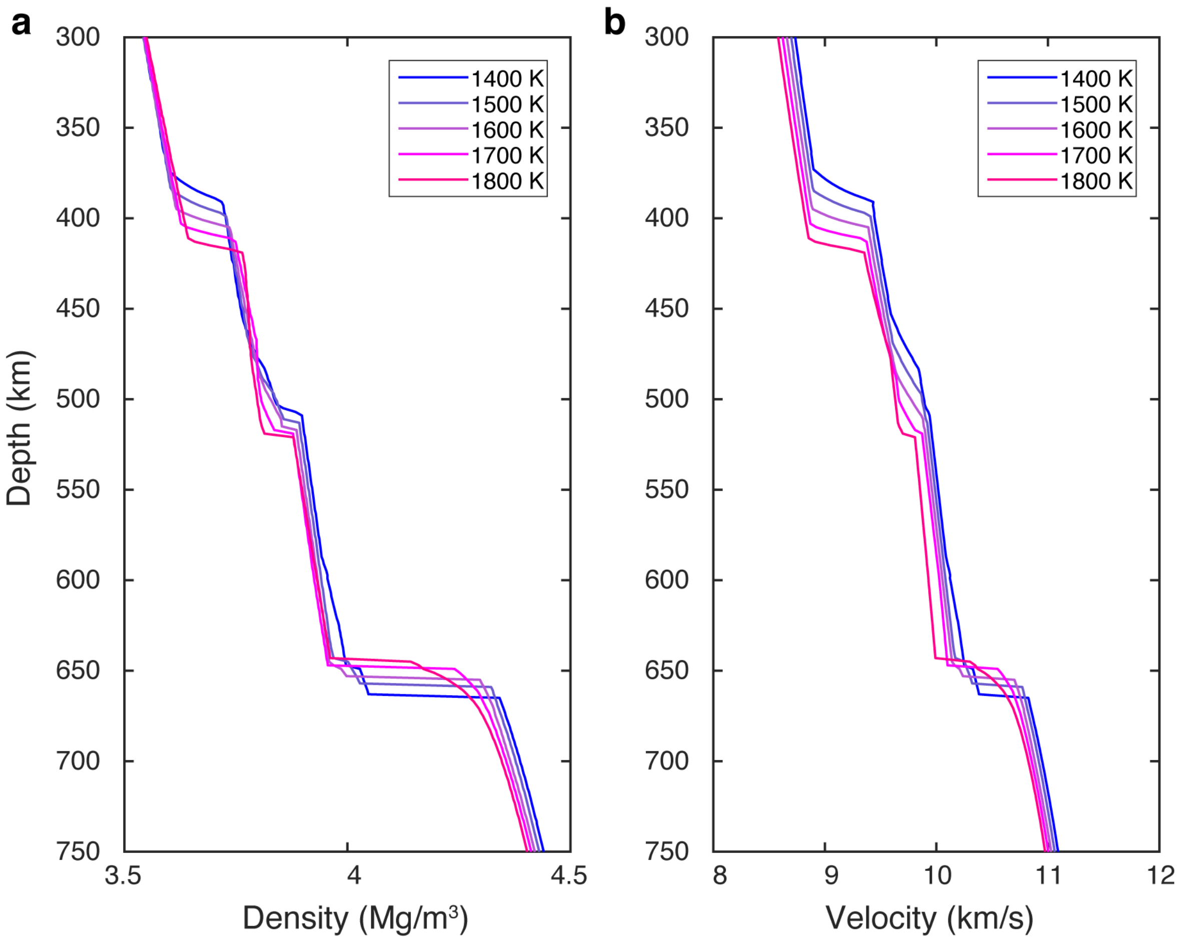

Supplementary Fig. 11. Seismic profiles calculated with different potential temperatures. **a** and **b** show the density and P-wave speed profiles for the pyrolite composition, respectively, calculated with different potential temperatures (1400 K, 1500 K, 1600 K, 1700 K, and 1800 K).

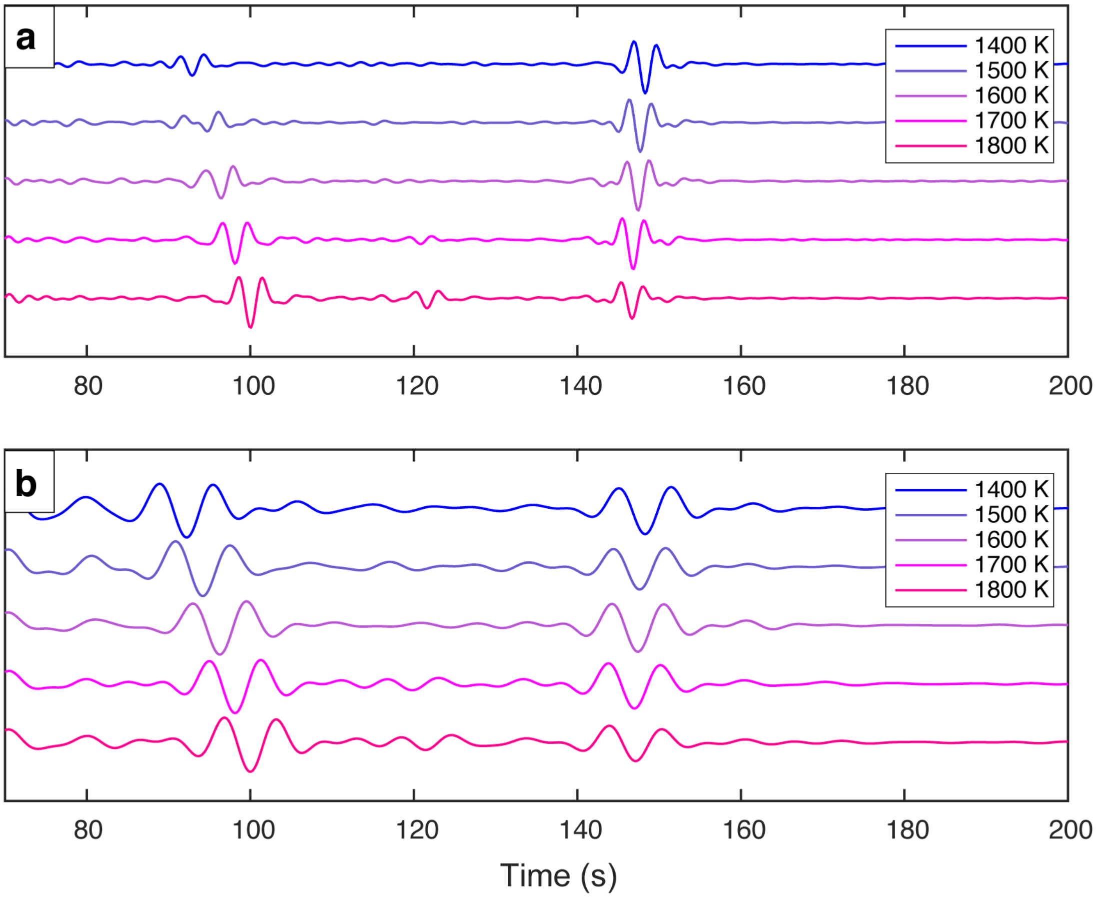

Supplementary Fig. 12. The influence of temperature on reflected waveforms. **a** Reflected body waves calculated from the seismic profiles of the pyrolite composition with different potential temperatures (1400 K, 1500 K, 1600 K, 1700 K, and 1800 K). All the waveforms are bandpass filtered to the 2-5 s period band and normalized within our target time window (80-200 s). **b** All the waveforms are bandpass filtered to the 5-10 s period band and subsequently normalized within the target time window.

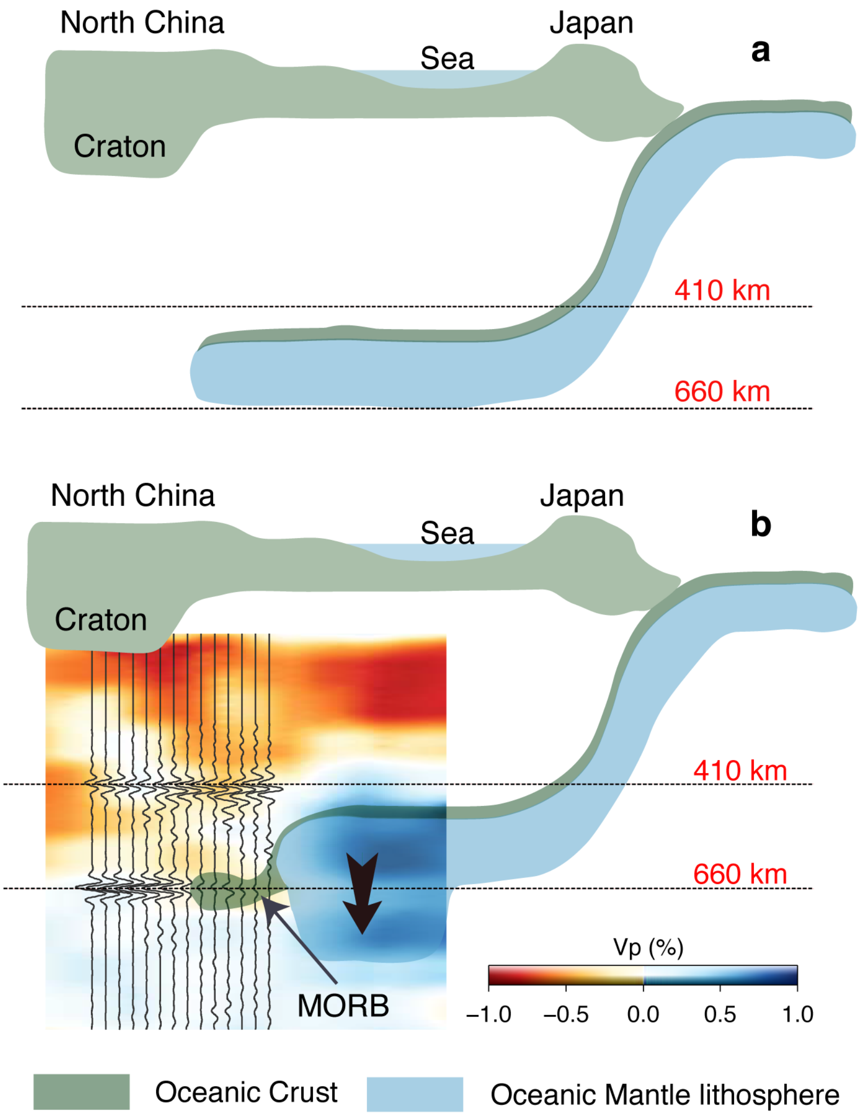

Supplementary Fig. 13. Schematic diagram for the evolution of the stagnant Pacific slab. **a** Subducted slab encounters resistance at the 660-km discontinuity, and the slab is deflected horizontally. **b** Part of the trapped slab finally sinks into the lower mantle and goes through composition segregation, producing a localized Mid-ocean-ridge basalts (MORB) enrichment layer at the base of the mantle transition zone beneath North China. Black traces and background color in **b** are the same as shown in Fig. 3b.

Supplementary Note 1

Description of the data set used in the study. We collected the continuous vertical component seismograms recorded by 200 stations from a dense portable seismic array and 208 permanent seismographs deployed within the North China Craton (NCC) (Fig. 1a). The time span is from October 2006 to July 2009 for the portable array and from July 2007 to December 2008 and the whole year of 2015 for the permanent stations. All collected waveform records were down-sampled at 5 Hz. The continuous seismograms of permanent stations for this study are archived at the Data Management Centre of China National Seismic Network at Institute of Geophysics, China Earthquake Administration (doi:10.11998/SeisDmc/SN, http://www.seisdmc.ac.cn). The waveform data of the portable array are archived at the China Seismic Array Data Management Center at Institute of Geophysics, China Earthquake Administration (doi:10.12001/ChinArray.Data, http://www.chinarraydmc.cn).

Supplementary Note 2

Slant stacking and comparison with theoretical values. In order to further confirm the observed phases, the raw ambient noise cross-correlation functions (NCFs) were converted from time-space domain to time-slowness domain by slant stacking[6]. Slant stacking was performed within several interstation distance bins: 0-200 km, 100-300 km, 200-400 km, and 300-500 km. The slant stacking results were compared with theoretical ray parameters and arrival times of P410P and P660P phases calculated with the Taup toolkit[1] from the ak135-f model[2] at epicentral distances of 100 km, 200 km, 300 km, and 400 km, respectively (Supplementary Fig. 3). The observed signals at ~100 s and ~150 s exhibit perfect agreement with P410P and P660P phases.

Supplementary Note 3

Common reflection point stacking results and resolution test. The linear and phase-weighted stacking results along three selected profiles are compared (Supplementary Fig. 5). For clarity, the waves reflected at shallower depths and the Rayleigh waves are tapered off. Generally speaking, the results from both stacking methods show great similarity, and both exhibit clear reflection signals from the 410- and 660-km interfaces. The phase-weighted stacking method can significantly improve signal-to-noise ratio by suppressing incoherent noise. The most significant feature of the reflected waveforms in the 5-10 s period band is the weak P660P near the high-speed Pacific slab. The weak P660P along profile AA' has already been reported in ref[7].

Here we design a targeted test to intuitively evaluate the resolution of the P660P phase to local gradual 660-km discontinuity, which is critical for our discussion in the main text. The designed 2-D models are derived from 1-D iasp91 model[4] with both sharp 410- and 660-km discontinuities. To avoid shallow reverberations within the crust, the velocity structure from the free surface to the Moho increases linearly. Local gradual 660-km discontinuity (a linear increase from 660 km to 720 km) is added to test the resolution of the P660P (Supplementary Fig. 6). Supplementary Fig. 7 shows the synthetic waveforms calculated with the SPECFEM2D package[5] from three models shown in Supplementary Fig. 6. Vertical force was set at 300 km on the free surface to excite the wavefield. No apparent frequency-dependence is observed for the body waves reflected from the sharp impendence contrasts at 410 and 660 km (Supplementary Fig. 7a, b). A 200-km wide gradual 660-km discontinuity induces remarkably weak P660P phase within two period bands (Supplementary Fig. 7c, d). On the other hand, a 100-km wide gradual 660-km discontinuity only generates very weak P660P phase within the shorter period band (2-5 s) (Supplementary Fig. 7e, f). It can be concluded from this test that a 200-km wide gradual 660-km discontinuity can be resolved by the P660P phase within the longer period band (5-10 s) and a 100-km wide gradual

660-km discontinuity can be resolved within the shorter period band (2-5 s).

Supplementary Note 4

Reliability test about the weak P660P phase. To check the reliability of the weak P660P phase, bootstrap test was performed within two example bins labeled A and B in Supplementary Fig. 8, respectively. Within the example bins, for each selected station pair, 20% of the daily NCFs were randomly selected and linearly stacked to obtain the final NCF for this station pair. Then all the NCFs of selected station pairs were linearly staked (the black trace in Supplementary Fig. 9a, b). The above processes were repeated 64 times within each bin to test the robustness of the reflected body waves. The stacked traces within bins A and B exhibit great robustness and the significant difference in amplitude ratio (P660P/P410P) looks reliable (Supplementary Fig. 9c).

To further validate the difference in the relative amplitude of P410P and P660P in the raw NCFs, we define two independent regions (Region A and Region B along the profile shown in Fig. 3) to show the stacked NCFs with respect to inter-station distance (Supplementary Fig. 10). As expected, coherent and clear P410P can be observed in both regions. However, clear and energetic P660P phases can only be observed in region A. This test validates the lateral variations in relative amplitude retrieved from NCFs.

Supplementary References

1. Crotwell, H. P., Owens, T. J. & Ritsema, J. The TauP Toolkit: Flexible seismic travel-time and ray-path utilities. *Seismological Research Letters* **70**, 154-160 (1999).
2. Kennett, B. L. N., Engdahl, E. R. & Buland, R. Constraints on Seismic Velocities in the Earth from Travel-Times. *Geophys J Int* **122**, 108-124 (1995).
3. Li, C., van der Hilst, R. D., Engdahl, E. R. & Burdick, S. A new global model for P wave speed variations in Earth's mantle. *Geochem Geophy Geosy* **9** (2008).
4. Kennett, B. L. N. & Engdahl, E. R. Traveltimes for Global Earthquake Location and Phase Identification. *Geophys J Int* **105**, 429-465 (1991).
5. Tromp, J., Komatitsch, D. & Liu, Q. Y. Spectral-element and adjoint methods in seismology. *Commun Comput Phys* **3**, 1-32 (2008).
6. Rost, S. & Thomas, C. Array seismology: Methods and applications. *Rev Geophys* **40**, doi:10.1029/2000rg000100 (2002).
7. Feng, J. *et al.* Depth variations of 410 km and 660 km discontinuities in eastern North China Craton revealed by ambient noise interferometry. *Geophys Res Lett* **44**, 8328-8335 (2017).
